# Design and Implementation of an Integrated Control System for Omnidirectional Mobile Robots in Industrial Logistics

**DOI:** 10.3390/s23063184

**Published:** 2023-03-16

**Authors:** Ahmed Neaz, Sunyeop Lee, Kanghyun Nam

**Affiliations:** School of Mechanical Engineering, Yeungnam University, 280 Daehak-Ro, Gyeongsan 38541, Republic of Korea

**Keywords:** ROS and LabVIEW interaction 1, autonomous robot 2, navigation with ROS 3, control design with ROS 4, SLAM 5, navigation with ROS, omnidirectional mobile robot 6, integrated control system 7, industrial logistic robots 8

## Abstract

The integration of intelligent robots in industrial production processes has the potential to significantly enhance efficiency and reduce human adversity. However, for such robots to effectively operate within human environments, it is critical that they possess an adequate understanding of their surroundings and are able to navigate through narrow aisles while avoiding both stationary and moving obstacles. In this research study, an omnidirectional automotive mobile robot has been designed for the purpose of performing industrial logistics tasks within heavy traffic and dynamic environments. A control system has been developed, which incorporates both high-level and low-level algorithms, and a graphical interface has been introduced for each control system. A highly efficient micro-controller, namely myRIO, has been utilized as the low-level computer to control the motors with an appropriate level of accuracy and robustness. Additionally, a Raspberry Pi 4, in conjunction with a remote PC, has been utilized for high-level decision making, such as mapping the experimental environment, path planning, and localization, through the utilization of multiple Lidar sensors, IMU, and odometry data generated by wheel encoders. In terms of software programming, LabVIEW has been employed for the low-level computer, and the Robot Operating System (ROS) has been utilized for the design of the higher-level software architecture. The proposed techniques discussed in this paper provide a solution for the development of medium- and large-category omnidirectional mobile robots with autonomous navigation and mapping capabilities.

## 1. Introduction

The COVID-19 pandemic has presented the global community with a unique challenge, and the scientific community has been working diligently to protect human health and maintain societal and industrial progress. The field of robotics has played a crucial role in this context. The utilization of different types of robots has been a highly researched topic in the wake of the pandemic. In fact, a survey [1] conducted in 2020 found that over 3500 papers were published on the topic of robots in contagion scenarios. Furthermore, the most significant research keywords, based on 280 publications, were mapped, with “autonomous robot” being among the top keywords. During the pandemic, the world has witnessed the successful deployment of robotic nurses [2] in Hong Kong, delivery robots in the United States, and working robots in Japan and Korea. Additionally, a study published in 2020 [3] indicates that since the onset of the COVID-19 pandemic, consumers are willing to pay an extra 61.28% for robot delivery.

The widespread adoption of robots has broadened the spectrum of human–robot collaboration, leading to an improvement in task accuracy and proximity to human employees. Among the various types of robots, mobile robots have gained significant attention for both industrial and logistic uses. The incorporation of autonomous robots in large-scale factories and logistics centers has become a common practice for reducing the strain on human labor.

For many years, autonomous guided vehicles (AGVs) [4] have dominated the robot industry due to their efficiency in handling manufacturing processes and logistics tasks, such as picking, packing, and palletizing, along pre-defined pathways. However, their inflexibility in adjusting to route changes and limited ability to collaborate with other systems or human operators has led to the development of a more advanced technology: autonomous mobile robots (AMRs) [5]. These robots have the capability of decision-making and autonomous navigation, without being restricted to a pre-defined path.

Another crucial consideration in terms of the integration of robots into human environments is the requirement for a proper understanding of the surrounding environment to avoid obstacles and unexpected encounters with humans or other objects. In fast-growing industrial environments with high traffic and narrow hallways surrounded by various objects and people, omnidirectional mobile robots (OMRs) [6] may be a superior solution due to their ability to move in any direction. However, their overlooked lower-level control design may not be effective in handling continuously changing loads. Thus, advanced control design, even for the lower-level control, is necessary to ensure the effectiveness of OMRs in heavy logistics duties.

In this research study, a design for a mobile robot has been proposed, featuring four Mecanum wheels driven by a bridge motor driver and controlled by a myRIO microprocessor. The rotation speed of these wheels allows for control over the forward, backward, and sideways movements, as well as the turning, of the robot. This research focused on studying different research and ideas from different projects and putting those puzzles together to create an improved and better-performing autonomous mobile robot.

The study aimed to develop a closed-loop feedback control system that incorporated both feedforward and Disturbance Observer (Dob) [7] with a graphical interface. The upper computer software was designed to enable remote control and monitoring of the robot, as well as to provide a user-friendly human–computer interaction.

Automatic navigation and mapping were performed using the Robot Operating System (ROS), which provided a Navigation Stack or Automatic Navigation System. This 2D or 3D [8] method integrates information from odometry, sensor data, and a goal pose to produce safe velocity commands. The Navigation Stack can generate the shortest path and avoid obstacles, even if those obstacles are not predetermined in the map data.

In order to build a map of the environment, Simultaneous Localization and Mapping (SLAM) was utilized. The G-Mapping [9] Package was employed for the robot, utilizing multiple LiDAR and odometry data and employing graph-based optimization to generate a highly accurate representation of the environment.

## 2. Designing Hardware Architecture

The design and construction of an autonomous robot involves a holistic consideration of both its mechanical and electrical components. This integrated approach is critical in ensuring that the robot functions optimally and efficiently in fulfilling its intended tasks. The developed robot was named “Motion Bot” and its mechanical and electrical components are thoroughly described in the subsequent sections of this paper. The comprehensive analysis of the mechanical and electrical components plays a critical role in illuminating the intricacies and interdependencies of the various elements that comprise the autonomous robot’s architecture.

### 2.1. Mechanical Components Design

**Figure 1:**
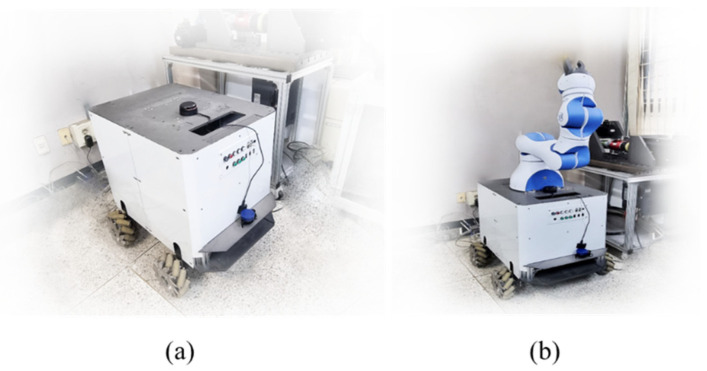
Appearance of Motion Bot (Experimental Robot) (**a**) without robotic arm; (**b**) with robotic arm.

The autonomous robot is designed with a lightweight aluminum body suitable for indoor environments. The design of the robot’s body was created using computer-aided design (CAD) software, which was utilized to perform simulations to calculate the load-bearing capacity of the robot. Upon successful design, the chassis was manufactured using a computer numerical control (CNC) machine. Figure 1 depicts the actual physical appearance of the robot.

Mobile robots equipped with non-holonomic systems possess the ability to move in a variety of directions regarding their current positions and orientations. This feature, known as omnidirectionality, is highly sought after in the field of mobile robotics. Several types of omnidirectional wheels exist, each with their own distinct advantages and disadvantages. The most common types of omnidirectional drives are the Kiwi and Holonomic systems [10], which require a precise arrangement to achieve omnidirectional motion. However, these wheels are not suitable for climbing ramps and have a lower capacity (approximately 50%) [11] for multi-directional movement. In contrast, Mecanum wheels, invented by Bengt Ilon, are highly efficient for both forward and reverse movements, as well as lateral movements. The orientation of Mecanum wheels can be arranged in a conventional manner, with lateral motion achieved through wheel velocity control.

In the current research, “Motion Bot” was equipped with four Mecanum wheels with a 100 cm diameter each, with twelve internal rollers at a 45-degree angle with the Y axis of the wheel. The wheels were connected to the main body frame via a suspension mechanism that provides surface contact conformity and reduces vibrations on the robot body.

**Figure 2:**
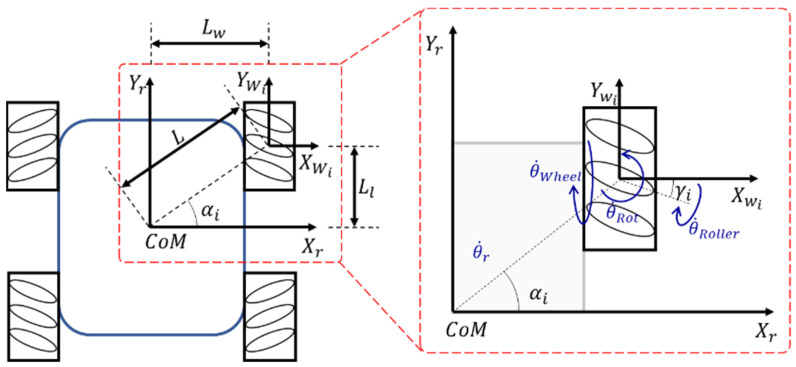
Direction of speed vector on Robot and Mecanum wheel.

Figure 2 presents a visual representation of the kinematic vector direction of the chassis, which incorporates the Mecanum wheel and its internal rollers. The procedure for determining the kinematics [12] of the system involves first calculating the inverse kinematics, and then calculating the pseudo-inverse [13]. This was achieved by utilizing a Cartesian coordinate system, which facilitated the analysis of vectors and other relevant variables. The list of variables and their definitions are listed in Table 1 also list of all symbols used in this article is expressed in Appendix A section.

To derive the kinematic equation, first, the relation between wheel velocity and the vehicle velocity was studied:

(1) $${\dot{X}}_{Wi}={\dot{X}}_{r}+{\dot{\theta }}_{r}\cdot L\cdot \cos\left(\frac{\pi }{2}+\alpha_{I}\right)$$

(2) $${\dot{Y}}_{Wi}={\dot{Y}}_{r}+{\dot{\theta }}_{r}\cdot L\cdot \sin\left(\frac{\pi }{2}+\alpha_{i}\right)$$

(3) $${\dot{\theta }}_{Wi}={\dot{\theta }}_{r}$$

Additionally, the relation between wheel velocity and the roller velocity was found:

(4) $${\dot{X}}_{Wi}=r\cdot {\dot{\theta }}_{Roller}\cdot \cos\left(\frac{\pi }{2}+\gamma_{i}\right)$$

(5) $${\dot{Y}}_{Wi}=r\cdot {\dot{\theta }}_{Roller}\cdot \sin\left(\frac{\pi }{2}+\gamma_{i}\right)+R\cdot {\dot{\theta }}_{Wheel}$$

(6) $${\dot{\theta }}_{Wi}={\dot{\theta }}_{Rot}$$

Now arranging Equations (1)–(3) in martrix form it can be written:

(7) $$\left[\begin{array}{c} {\dot{X}}_{Wi}\\ {\dot{Y}}_{Wi}\\ {\dot{\theta }}_{Wi} \end{array}\right]=\left[\begin{array}{ccc} 1 & 0 & L\cdot cos(\frac{\pi }{2}+\alpha_{i})\\ 0 & 1 & L\cdot sin(\frac{\pi }{2}+\alpha_{i})\\ 0 & 0 & 1 \end{array}\right]\left[\begin{array}{c} {\dot{X}}_{r}\\ {\dot{Y}}_{r}\\ {\dot{\theta }}_{r} \end{array}\right]$$

and arranging Equations (4)–(6) in matrix form:

(8) $$\left[\begin{array}{c} {\dot{X}}_{Wi}\\ {\dot{Y}}_{Wi}\\ {\dot{\theta }}_{Wi} \end{array}\right]=\left[\begin{array}{ccc} 0 & r\cdot cos(\frac{\pi }{2}+\gamma_{i}) & 0\\ R & r\cdot sin(\frac{\pi }{2}+\gamma_{i}) & 0\\ 0 & 0 & 1 \end{array}\right]\left[\begin{array}{c} {\dot{\theta }}_{Wheel}\\ {\dot{\theta }}_{Roller}\\ {\dot{\theta }}_{Rot} \end{array}\right]$$

From Equations (7) and (8) it can be written:

(9) $$\left[\begin{array}{c} {\dot{\theta }}_{Wheel_{i}}\\ {\dot{\theta }}_{Roller_{i}}\\ {\dot{\theta }}_{Rot_{i}} \end{array}\right]=\left[\begin{array}{ccc} \frac{1}{R\cdot tan(\gamma_{i})} & \frac{1}{R} & \frac{L}{R}(\cos\left(\alpha_{i}\right)-\sin\left(\alpha_{i}\right)\cdot \cot\left(\gamma_{i}\right))\\ -\frac{1}{r\cdot sin(\gamma_{i})} & 0 & L\cdot \frac{\sin\left(\alpha_{i}\right)}{r\cdot sin(\gamma_{i})}\\ 0 & 0 & 1 \end{array}\right]\left[\begin{array}{c} {\dot{X}}_{r}\\ {\dot{Y}}_{r}\\ {\dot{\theta }}_{r} \end{array}\right]$$

Within the context of Equation (9), the angular velocity of the roller is not a focal point of consideration as the wheels are securely attached to the motor, thereby eliminating any potential for rotational velocity in the yaw direction. Hence, through considering the angular velocity of the wheel, the conclusion can be:

(10) $${\dot{\theta }}_{Wheel_{i}}=\frac{1}{R\cdot tan(\gamma_{i})}\cdot {\dot{X}}_{r}+\frac{1}{R}\cdot {\dot{Y}}_{r}+\frac{L}{R}(cos(\alpha_{i})-\sin\left(\alpha_{i}\right)\cdot \cot\left(\gamma_{i}\right))\cdot {\dot{\theta }}_{r}$$

Table 2 is listed with the wheel and roller angular parameters for each wheel of the experimental robot.

Substituting the $I\;and\;I_{i}$ value in Equation (10), we can rewrite the equation as it is written below:

(11) $$\left[\begin{array}{c} {\dot{\theta }}_{Wheel_{1}}\\ {\dot{\theta }}_{Wheel_{2}}\\ {\dot{\theta }}_{Wheel_{3}}\\ {\dot{\theta }}_{Wheel_{4}} \end{array}\right]=\frac{1}{R}\left[\begin{array}{ccc} -1 & 1 & \left(L_{l}+L_{w})((cos(\alpha_{1})-\sin\left(\alpha_{1}\right)\right)\\ 1 & 1 & -(L_{l}+L_{w})((cos(\alpha_{2})-\sin\left(\alpha_{2}\right))\\ -1 & 1 & -(L_{l}+L_{w})((cos(\alpha_{3})-\sin\left(\alpha_{3}\right))\\ 1 & 1 & \left(L_{l}+L_{w})((cos(\alpha_{4})-\sin\left(\alpha_{4}\right)\right) \end{array}\right]\left[\begin{array}{c} {\dot{X}}_{r}\\ {\dot{Y}}_{r}\\ {\dot{\theta }}_{r} \end{array}\right]$$

Equation (11) is the inverse kinematics of the system, and to find the forward kinematics, the pseudo-inverse process of Equation (11) must be processed, and then the equation will be:

(12) $$\left[\begin{array}{c} {\dot{X}}_{r}\\ {\dot{Y}}_{r}\\ {\dot{\theta }}_{r} \end{array}\right]=\frac{R}{4}*\;\left[\begin{array}{cccc} -1 & 1 & -1 & 1\\ 1 & 1 & 1 & 1\\ \frac{1}{\left(L_{l}+L_{w})((cos(\alpha_{1})-\sin\left(\alpha_{1}\right)\right)} & \frac{-1}{\left(L_{l}+L_{w})((cos(\alpha_{2})-\sin\left(\alpha_{2}\right)\right)} & \frac{-1}{{(L}_{l}+L_{w})((cos(\alpha_{3})-\sin\left(\alpha_{3}\right))} & \frac{1}{{(L}_{l}+L_{w})((cos(\alpha_{4})-\sin\left(\alpha_{4}\right))} \end{array}\right]*\;\left[\begin{array}{c} {\dot{\theta }}_{Wheel_{1}}\\ {\dot{\theta }}_{Wheel_{2}}\\ {\dot{\theta }}_{Wheel_{3}}\\ {\dot{\theta }}_{Wheel_{4}} \end{array}\right]$$

### 2.2. Hardware Connection and Configuration

**Figure 3:**
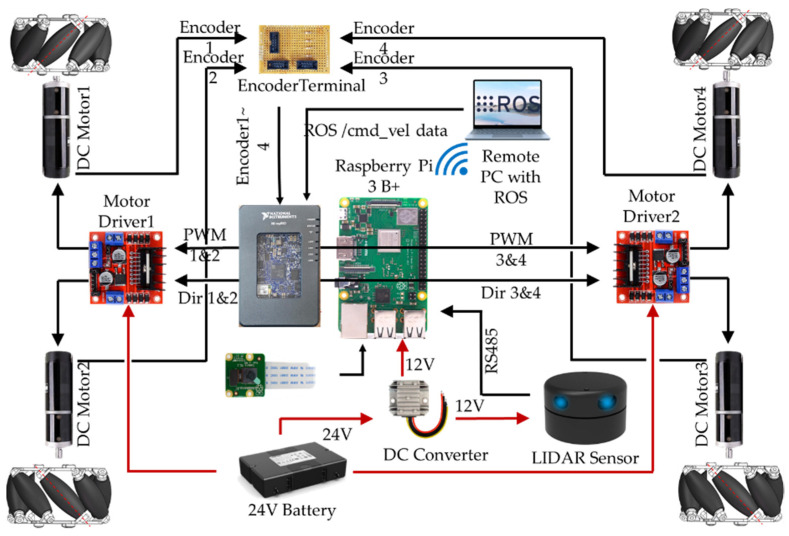
Connection Diagram of Different electrical components.

For the experimental robot divide, the electrical components were divided into three classes. The first one is the decision-making and control components, the second one is the sensors, and the last one is the power system. Figure 3 shows the hardware connection of all mobile robot parts, where remote PC is the upper computer base. The ROS master is executed from here, which sends all the control instructions using a common Wi-Fi signal channel. Raspberry Pi works as a second upper computer base that collects data from LiDAR and camera sensors. MyRIO works as the main controller, which receives control instructions from the upper computer base through Wi-Fi to control the DC (Direct Current) motors through the bridge driver, as well as send encoder data sets as a ROS node. For the power source of the robot, a battery of 24 V was used with BMS (Battery Management system) and the power carrying capacity was 12 Ah. A 24 V to 12 V DC to DC converter is used here, as the motors’ running voltage is 24 V, but myRIO and Raspberry Pi can operate with a 12 V maximum power supply.

**Figure 4:**
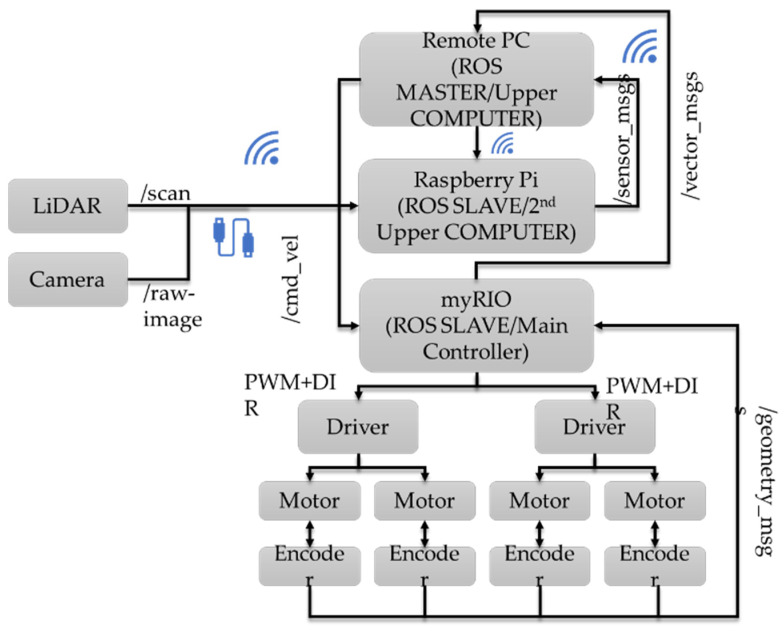
Visualization of Data flow.

It is acknowledged that utilizing a single upper computer, such as Raspberry Pi, for processing heavy data may result in a decrease in performance. To ensure efficient monitoring and prompt response, a remote PC is utilized in conjunction with Raspberry Pi. Figure 4 shows the data flow within this connection mentioning the ROS topic name.

## 3. Designing Software Architecture

The software architecture design will concentrate on the creation of velocity control mechanisms for the motors, the mapping of the surrounding environment, and the implementation of an autonomous navigation system. The control architecture has been bifurcated into two sections for comprehensive elucidation. The first component deals with the velocity control, which is referred to as the lower-level control and is exclusively accountable for executing directives without any decision-making capacity. Conversely, the higher-level control imbues the robot with the capacity to perceive its environment, generate trajectories towards a designated target, and make adaptive choices for obstacle avoidance.

### 3.1. Lower-Level Control Software Design

The control design of a mobile robot can be approached from either a dynamic or a kinematic perspective. While the dynamic approach involves the calculation of the real-time system and is more complex, the kinematic approach, which consists of both the kinematic loop and dynamics loop, is simpler and can guarantee stability through proper tuning. This study adopts the kinematic approach for the control design and classifies it into four sections. The first section focuses on finding the system identification and establishing a nominal model, followed by the feedback control loop, along with the feedforward and disturbance observer, in the second section. The third section addresses the design of various trajectories to evaluate the control performance, and the final section analyzes the robustness of the closed-loop system. LabVIEW programming was utilized for the lower-level control, providing a Human Machine Interface (HMI) that allows for real-time adjustment of control parameters and the creation of trajectories for automated guided robots.

#### 3.1.1. System Identification

**Figure 5:**
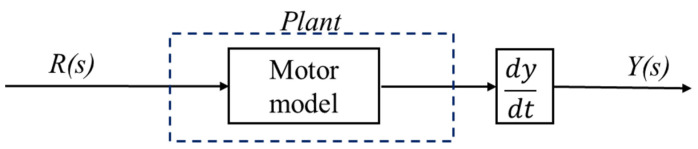
System Identification process block diagram.

Since electrical components, such as motor resistance and inductance, are controlled by the motor driver, we will focus on the mechanical parts for system identification. The nominal model for each wheel was identified through this process. Figure 5 shows a block diagram of the process used for this process.

**Figure 6:**
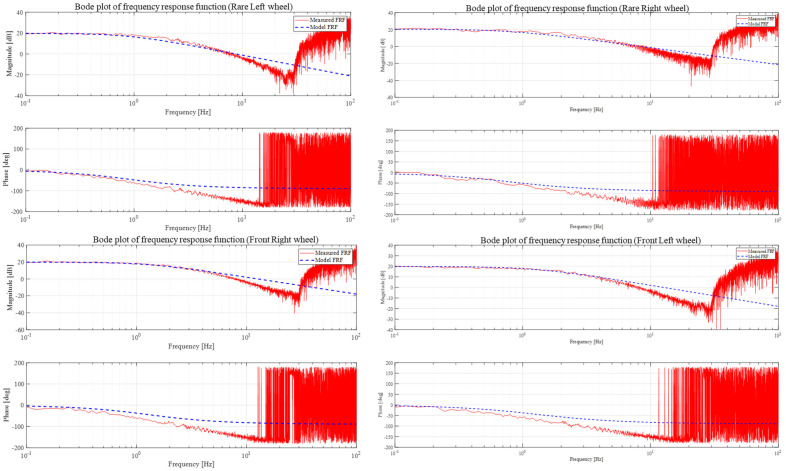
Bode plot Diagram.

For system identification [14] of four wheels, a chirp sine signal of 0~10 Hz was applied for 10 s. PWM value was 0~1%, and the sine magnitude was 0.7, 0.75, 0.8, and 0.85. Figure 6 shows the body plot diagram of model design.

As it can be seen from the body plot, the magnitude has dropped around 20 dB during 1 log-based frequency change, so we can be assured that the system model is the 1st order [15] and that the mathematical form of the nominal model should be:

(13) $$\frac{Output}{Input}=\frac{1}{J_{n}s+B_{n}}$$

#### 3.1.2. Control Design

The method for motor control [4] used in this experiment was speed-voltage looped control. Voltage was considered equivalent to velocity, and control was designed for each individual motor. Then, from the forward kinematics on Equation (12), we can calculate the individual motor’s velocity to find the total vehicle velocity. The actual velocity provided by each motor encoder can be calculated using Equation (11). Then, using the given velocity and actual velocity, a feedback control loop can be designed. Using the nominal model from Section 3.1.1, a feedback control loop was designed through pole-zero cancelation method [16]. The feedback control equation design was as follows:

(14) $$C_{fb}=\omega_{fb}\cdot J_{n}+\frac{\omega_{fb}\cdot B_{n}}{s}\;(Here,\;\omega_{fb}=2\pi \times 2\;Hz)$$

To soothe the loading torque on the DC motor speed and make the response time fast, feedforward compensation was designed by taking the inverse of the nominal model and multiplying it with a low-pass filter. The feedforward control equation for this robot was as follows:

(15) $$C_{ff}=\frac{\left(J_{n}s+B_{n}\right)}{\frac{s}{\omega_{ff}}+1}\;(Here,\;\omega_{ff}=2\pi \times 10\;Hz)$$

Even though the use of both feedback and feedforward control were adequate for operating under no-load conditions, there was a noticeable degradation in the control system’s performance under varying loads. Furthermore, it was necessary to consider model uncertainty. To mitigate this issue, a disturbance observer was incorporated. This addition will address system disturbances, as well as sensor noise, thereby leading to an enhanced control system performance. For designing a disturbance observer, we have used the inverse of our nominal model with a Q filter. The equation for the Q filter was as follows:

(16) $$Q(s)=\frac{\omega_{Q}^{2}}{s^{2}+2\zeta \omega_{Q}s+\omega_{Q}^{2}}\;(Here,\;\omega_{Q}=2\pi \times 2\;Hz)$$

**Figure 7:**
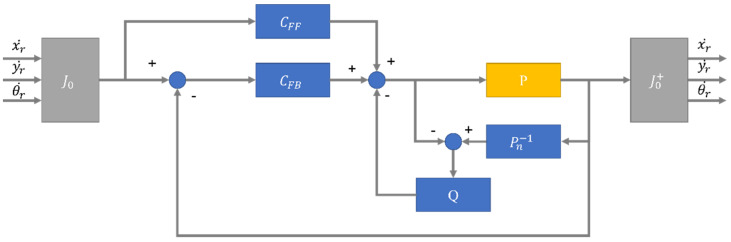
Block Diagram of control algorithm.

Figure 7 shows a block diagram of the control algorithm, where $\dot{x_{r}},\;\dot{y_{r}},\;{and\;\theta }_{r}$ are linear x, linear y, and the angular velocity of the robot, and they are controlled with a feedforward and a feedback loop, along with a disturbance observer. The list of symbols used in Figure 7 and there meanings are listed in Table 3. A study using such kind of control algorithms is conducted in a journal by Mu-Tian Yan and Yau-Jung Shiu [17], and it was established that this kind of control strategy was adequate for controlling motors.

#### 3.1.3. Control Performance Test

**Figure 8:**
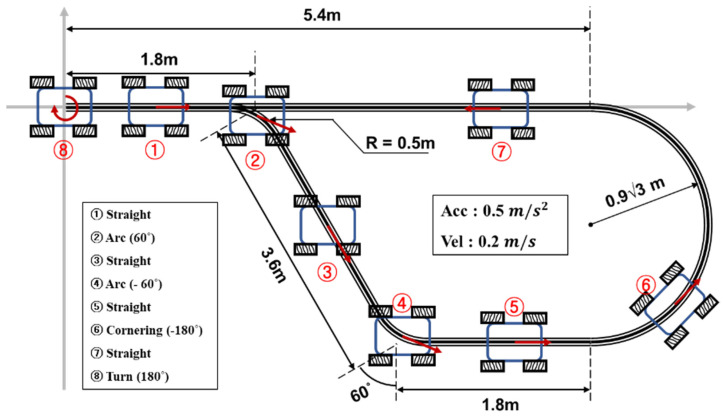
Trajectory of the experimental robot.

The performance evaluation of the lower-level control was conducted using a trajectory similar to the one shown in Figure 8. The trajectory incorporated straight motion, arc cornering, and turning motion with varying velocity for the purpose of testing. Data collection was performed utilizing the USATR (Universal Synchronous/Asynchronous Receiver/Transmitter) method [18], and the results were plotted using MATLAB. The velocity data was calculated directly from the kinematics, while the position data was obtained through the application of the discrete time integration method on the velocity data.

**Figure 9:**
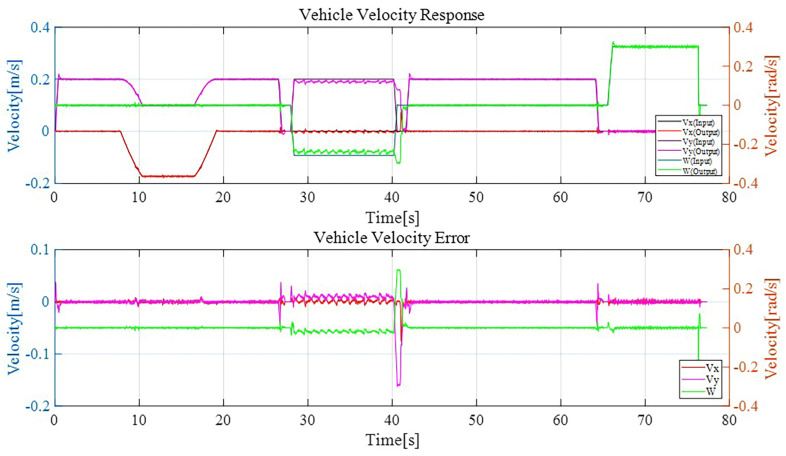
Velocity input vs. output plot.

In Figure 9, the velocity plot and velocity error plot have been shown to follow the guided trajectory. Here, $V_{x},\;V_{y},\;and\;W$ are longitudinal, lateral, and angular velocity, respectively.

From the error plot, it can be clearly seen that the velocity error is below 0.05 m/s on average. There is some overshoot on certain positions, but the overall system is stable and there is no steady state error.

**Figure 10:**
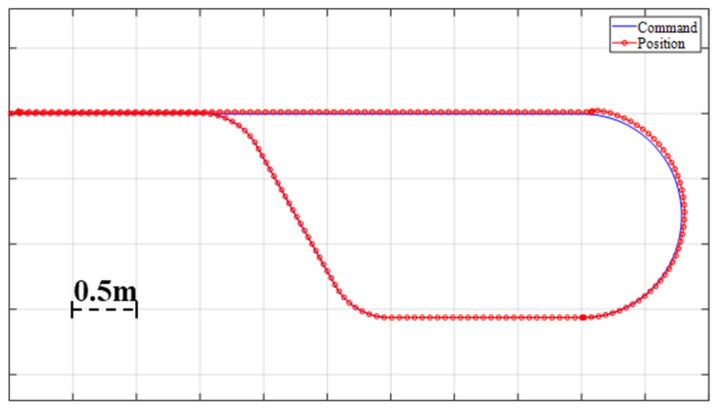
Position input vs. output plot.

Figure 10 displays a plot of the commanded position and the actual position, as calculated by the motor encoder. The plot demonstrates that the robot is capable of following the command effectively while traversing straight motion and cornering. However, a negligible error, due to overshoot, is observed during the turning motion. During the evaluation of the lower-level control, the possibility of wheel slip was not taken into account, as it is addressed during the design phase of the higher-level control.

#### 3.1.4. Robust Performance Test

In this section, the robustness of the designed control system based on the disturbance observer (DOB) will be analyzed [19]. To analyze robustness, the selection of system uncertainty was studied first. The system uncertainty ±30% of the nominal model for both inertia $\left(J_{n}\right)$ and friction ($B_{n}$) was selected and analyzed.

Next, uncertainty weight selection was conducted through the following equations. To describe the generic model uncertainty with a complex norm-bounded multiplicative uncertainty, the equation is:

(17) $$P\left(s\right)=\left(1+W_{2}\left(s\right)∆\left(s\right)\right)P_{n}\left(s\right)\;where,\;{\left\|∆\left(s\right)\right\|}_{\propto }\leq 1$$

The weight $W_{2}\left(s\right)\;is\;selected\;so\;that$:

(18) $$\underset{P\in P}{\max}\left|\frac{P\left(j\omega \right)-P_{n}\left(j\omega \right)}{P_{n}\left(j\omega \right)}\right|\leq \left|W_{2}\left(j\omega \right)\right|$$

Here, a set of perturbed plant models P is obtained by varying the values of J and B within their variability ranges:

(19) $$P=\left\{P\left(s\right)=\frac{1}{Js+B}\;[Here,J=J_{n}\pm 30\%,\;B=B_{n}\pm 30\%]\right\}$$

Now, the driven equation is as follows:

(20) $$\frac{P\left(j\omega \right)-P_{n}\left(j\omega \right)}{P_{n}\left(j\omega \right)}=\frac{\frac{1}{Js+B}-\frac{1}{J_{n}s+B_{n}}}{\frac{1}{J_{n}s+B_{n}}}\times \frac{\left(Js+B\right)\left(J_{n}s+B_{n}\right)}{\left(Js+B\right)\left(J_{n}s+B_{n}\right)}=\frac{\left(J_{n}s+B_{n}\right)-\left(Js+B\right)}{Js+B}\;=\frac{\left(J_{n}-J\right)s+\left(B_{n}-B\right)}{Js+B}$$

**Figure 11:**
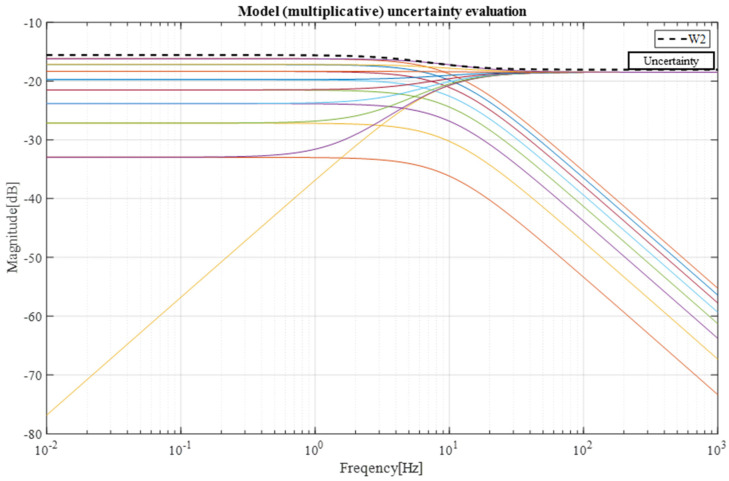
Uncertainty weight selection.

Figure 11 shows the selection of uncertainty weight function and its bode plot.

Here, uncertainty weight is selected as:

(21) $$W_{2}=K*\frac{1+\frac{s}{\omega_{z}}}{1+\frac{s}{\omega_{p}}}\left[here,\;\omega_{z}=2*\pi *8,\;\omega_{p}=2*\pi *6,\;K=0.125\right]$$

**Figure 12:**
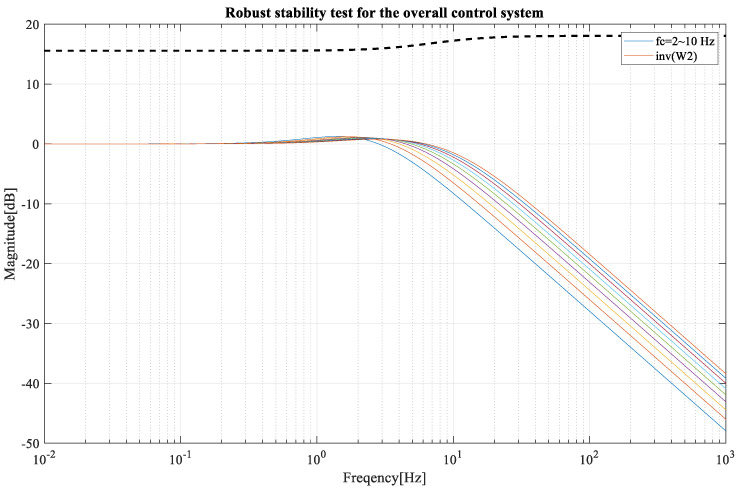
Robust stability for overall system.

The robust stability for the overall system follows $T^{\prime }=\frac{P_{n}C+Q}{1+P_{n}C}$, which is shown in Figure 12 for a feedback cutoff frequency from 2 to 10 Hz.

### 3.2. Higher-Level Control Software Design

The higher-level controller plays a crucial role in ensuring the efficient and safe operation of the robot by generating a reference path that avoids potential collisions. This is achieved through the creation of a map of the environment that localizes the robot within it. The software utilized by the upper computer is based on the Robot Operating System (ROS), which serves as a framework for programming hardware components such as motors, sensors, and drivers.

ROS supports multiple programming languages, including C++, Python, and Java, and allows for the use of multiple programming languages across multiple connected computers. Additionally, ROS is capable of executing multiple executables in parallel, allowing for both synchronous and asynchronous data exchange between them. These executables, referred to as ROS nodes, share data through ROS topics.

ROS also provides graphical interfaces, such as RVIZ [20], from which we can visualize all the sensor data and related values in real time. ROS also comes with SLAM and Navigation stack packages, which have the adequate processes to make a perfect map of the environment and navigate it with safety. For designing the higher-level control software, the ‘turtulebot3′ [21] and ‘Nox’ [22] package structures were used with modification needed for our experimental robot. Additionally, as three LiDAR sensors were installed, we used a lidar merger package to combine those scan data.

#### 3.2.1. ROS Package Modification

For architecting the higher-level software, several suitable modifications were performed, the most notable of which was the odometry package modification. As robots can also move in the lateral direction, a calculation was needed to consider this motion. Additionally, to use mechanomes we must consider the pose error due to slip ratio. To overcome this, we used the pose created by the wheel encoder data and made an estimated odometry using sensor fusion of the lidar sensor, IMU, and encoder data.

#### 3.2.2. Connection of Higher and Lower Software

For this experiment, NI myRIO was used for lower-level control and collecting odometry data, which can be programmed by NI LabVIEW software. LabVIEW provides an add-on named “ROS for LabVIEW,” which can be downloaded from the VI Package store. However, as ROS is operated mainly using the Ubuntu (Linux) system and LabVIEW software is mainly operated using the Windows system, we need to take several steps to connect these two systems. The preconditions to connect ROS with LabVIEW are:

1. All the Wi-Fi connections should be under the same network and the first 7 digits of the IP address have to be the same for all devices.
2. Host IP address should be added to both Ubuntu and Windows systems using Administrator’s access.
3. Accessibility of each device should be checked using the “ping” command.
4. The antivirus network protection should be off, or new protocols should be made for those IP addresses.
5. ROS Master IP address and ROS Host IP address should be set before running ROSCORE.

If Windows Firewall does not allow the ROS network to communicate LabVIEW, then Windows Firewall Rule should be made. The steps are:

1. Open Control Panel > System and Security > Windows Firewall > Advanced Settings
2. Right-click “Inbound Rules” and select “New Rule”
3. Assign the following properties to the new rule
  - Select “Custom Rule” under “Rule Type.”
  - Under the protocol and port for the protocol type, select “ICMPv4.”
  - Apply to all local and remote IP addresses in the range.
  - In terms of connections you are allowed to choose, check “Domain,” “Private,” and “Public” in Profile.
  - Assign a name, such as “ICMPv4 rule for ROS communication,” and choose “Finish.”

**Figure 13:**
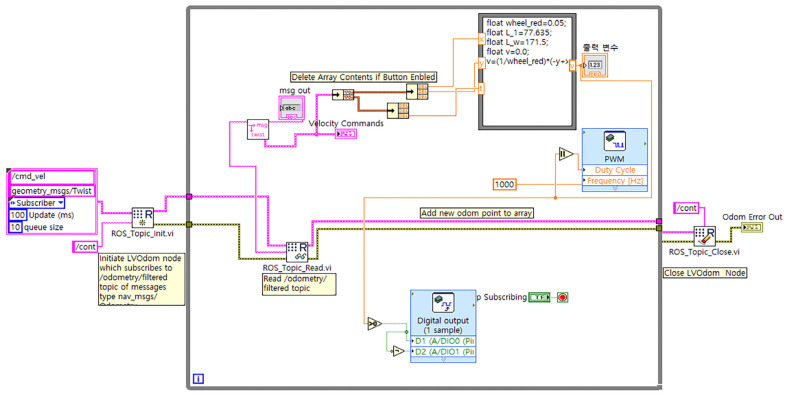
ROS Programming with LabVIEW (subscriber to cmd_vel topic).

After successful establishment of the ROS network, it is time to Run ROS on the LabVIEW system. Figure 13 shows a simple VI, which will subscribe to the /cmd_vel topic and read the twist message of Linear and Angular Velocity. Reading those messages from ROS, the LabView will execute Linear and Angular motion by running the motors through the myRIO device.

Before running the VI, we should double click ROS_Topic_init.vi and re-correct the topic name and message type if needed. It is always best practice to run the ROS Master inside LabVIEW to ensure the node is working fine. Otherwise, some errors can occur, and it will become harder to reconnect.

**Figure 14:**
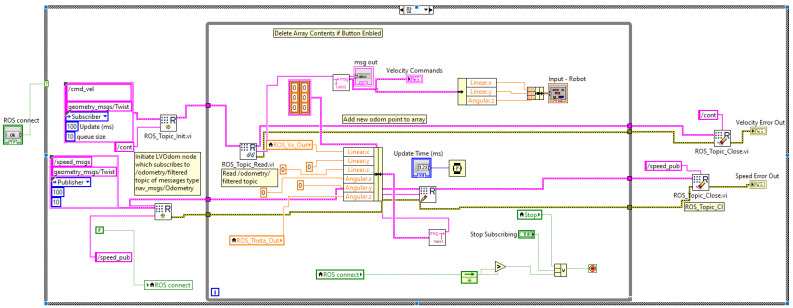
ROS Programming with LabVIEW (publisher and subscriber of different topic).

The complete software, Architecture, is also divided into several tasks, such as receiving velocity commands through a node from the Master Computer, processing the input velocity through control algorithms to match that and generate the PWM and direction signal for motor drivers, and lastly, calculating the velocity of the robot reading the encoder data and sending it to the ROS Master through another node. Figure 14 shows a program in LabVIEW where a subscriber node is created, which will receive velocity command, and another publisher node is created, which will publish the linear and angular velocity of the robot.

## 4. SLAM based on ROS

SLAM refers to the process of creating a map of an unknown environment while simultaneously determining the robot’s location within it. This is achieved through the use of sensors, such as lidar sensors or GPS, and wheel odometry. The task of simultaneously performing both localization and mapping presents a significant challenge, akin to navigating and mapping a large, unknown house. The SLAM algorithm is dependent on probabilistic models, which take into account uncertainty and estimation processes. Researchers from diverse fields are actively exploring ways to improve the representation of both the environment and the robot’s position. The advancement of various sensors has led to the widespread use of SLAM in various applications, including rescue operations, archaeology, and military and industrial contexts. One of the most widely used SLAM methods in the ROS framework is the GMapping algorithm. This method is based on the Rao–Blackwellized particle filter (RBPF) [23] and has proven to be highly effective in acquiring maps of unknown dynamic environments. Other popular SLAM algorithms, such as Hector SLAM and First SLAM, have unique uses and capabilities, but GMapping stands out for its ability to fuse multiple sensor data sources together using a Kalman filter [24] to achieve more accurate estimations.

**Figure 15:**
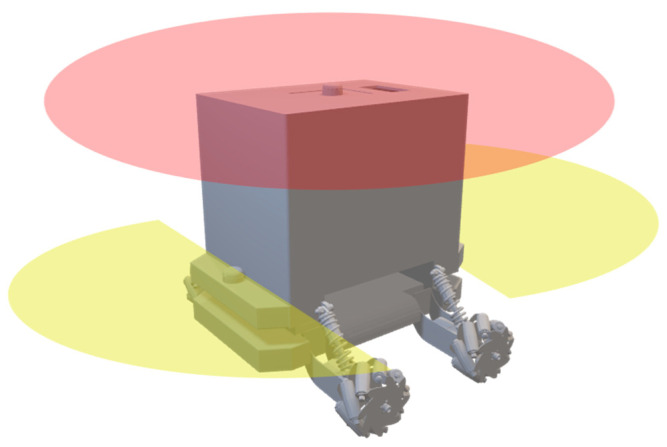
Position of LiDAR sensors and covering area.

To make a perfect SLAM, four sets of data are required. First, the robot’s position in both the steady and the moving condition is needed. For this experiment, the initial position was introduced to the robot. Second, sensing or measuring surrounded obstacles from the robot; this was carried out by using the LiDAR sensor. Third, the initial map of the robot, which can be made at the steady position of the robot, and fourth, a path by which the robot moves in that unknown environment, which was covered using odometry and IMU sensor data. However, as our robot is a medium-sized mobile robot, using only one LiDAR sensor is not enough. This is because if the LiDAR is installed only on the top, it cannot cover the area below that. To solve this problem, we have implemented three LiDAR sensors, shown in Figure 15. One LiDAR on the top will cover 360°, and the other two LiDAR on the front and back will cover 180° from the bottom. Merging them together will provide precise information about surrounding obstacles.

**Figure 16:**
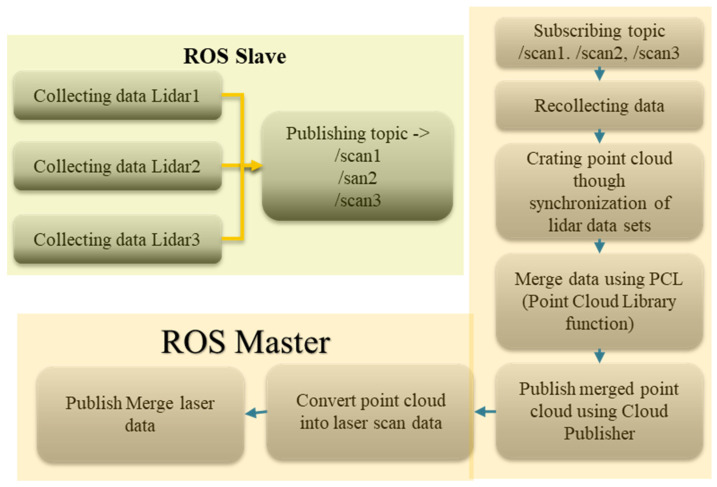
Algorithm for merging 3 lidar scan data for mapping.

To merge these three LiDAR data together, a ROS package was created by following different papers [25,26] related to multi-LiDAR sensor collaboration approaches. Figure 16 shows the algorithm used to merge three lidar sensor data and publish it as one laser data. In this algorithm, ROS slave on the Raspberry Pi board is responsible for collecting all data sets from three lidar sensors and publish it as a node with a different topic name for each individual LiDAR. Then, ROS Master, running on a laptop, will combine those topics and recollect those data sets. Then, through the synchronization of those data, a point cloud will be created. Then, we can merge data using the point cloud library and publish that merged point cloud data. After that process, we can convert the point cloud data into laser data and publish the merged laser data.

**Figure 17:**
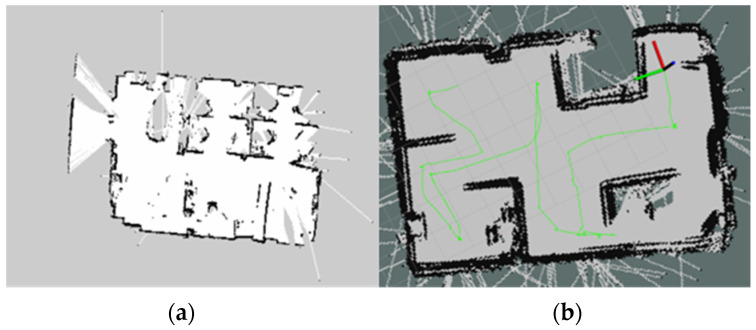
(**a**) SLAM performance with single Lidar; (**b**) SLAM performance with multiple Lidar and IMU.

Figure 17 shows the difference between performance of SLAM using single LiDAR and Multiple LiDAR. In Figure 17b, we can clearly see a better performance and clear map of the environment using multiple LiDAR. We can also see some errors which were mainly generated due to noise, and this can be reduced through further research and development. The green line in Figure 17b indicates the trajectory of the robot while making the map with the SLAM algorithm.

## 5. Navigation Based on ROS

**Figure 18:**
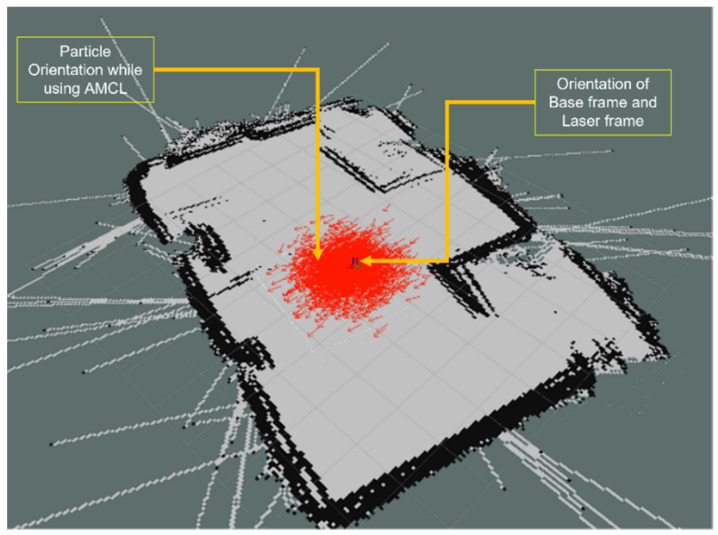
Monte Carlo Localization.

The Navigation Stack is a highly advanced package of ROS software, capable of performing both localization and autonomous navigation with a planned trajectory. This package is comprised of three sub-packages, including the Adaptive Monte Carlo Localization (AMCL) [27] module, which is responsible for localizing the robot within a map using particle filters and odometry and laser data. In its initial position, the upper computer has limited data to calculate the exact position of the robot, resulting in a large circular area. However, as the robot moves, the point cloud accumulates more data, allowing for a more accurate calculation of the robot’s position. Figure 18 shows an implementation of AMCL, where red arrows show the possible position of the robot within the map.

The second sub-package, the Map Server, is responsible for reading the map created by SLAM from disk storage and serving it as a topic named /map to the ROS master. The third sub-package, the Move Base package, is responsible for generating a secure and efficient path for autonomous navigation. This package reads various initial conditions, such as the robot’s footprint dimensions, obstacle range, and maximum and minimum linear and angular velocity, from YAML files. It then generates a path using algorithms [28] such as A-star, Rapidly-exploring Random Tree (RRT), or RRT Star, and various optimization techniques, such as Genetic Algorithm (GA), Artificial Intelligence (AI), and Particle Swarm Optimization (PSO).

**Figure 19:**
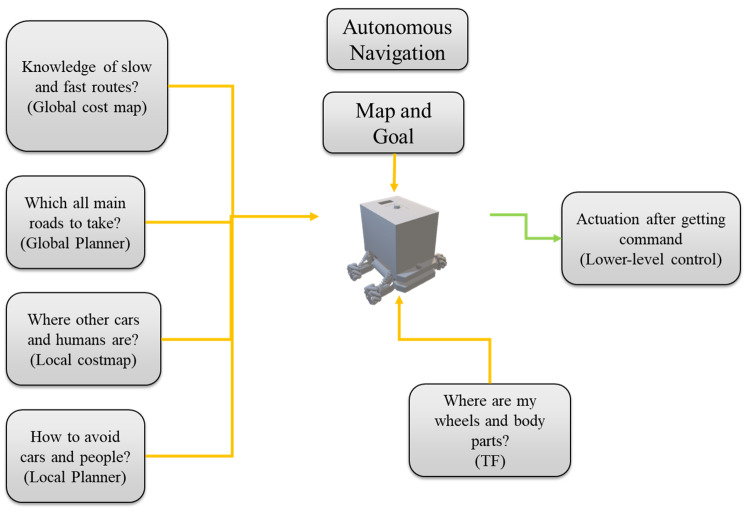
ROS Navigation Stack parts and their roles.

Figure 19 shows the roll of different ROS navigation stack files [29]. An important thing to note here is that the cost map is divided into a global cost map and a local cost map, where the global cost map contains overall information about the entire environment and the local cost map contains information about the surrounding obstacles of the robot. The global path planner is responsible for creating the main trajectory to reach the goal, while the local path planner is responsible for avoiding small obstacles by correcting the main trajectory generated by the global planner. The path planner algorithm used in this paper is adopted from a research study conducted by LIU Tianyu, YAN Ruixin, WEI Guangrui, and SUN Lei [29].

In order to perform autonomous navigation with the robot, modifications to the ROS navigation stack parameters were necessary to account for the specific dimensions and environment of the robot. A threshold of 350 mm was applied on the edge of obstacles to avoid collisions, and proper path planning was executed. In the event of new obstacles (e.g., a walking person) appearing in the path of the planned trajectory, which are not present in the global map, they are added to the local map and the move base package re-plans the path to reach the goal. Additionally, lateral path planning freedom was added by modifying various files in the ROS navigation stack.

## 6. Results and Discussion

In this section, results and analyses will be discussed to check accomplishments. Through that discussion, some issues and observations that were faced during testing the robot will be mentioned, and further research goals will be determined to take the robot to the next level. To best discuss the results, this section was divided into two sub-sections. In the first section, the lower-level control performance will be discussed, and in the second section, the higher-level control performance will be discussed.

### 6.1. Lower-Level Control Results

From the experiment result attached in Figure 9, it can be observed that the lower-level controller can perform with a gratifying accuracy. In the position plot in Figure 10, the error was less than 0.05. Through the robustness analysis, we found that both the disturbance observer loop and overall control loop were under the curve of uncertainty, weighting the function magnitude line shown in Figure 11 and Figure 12. Thus, theoretically, both of the loops were stable, which means that even if we added 30% more load than expected, the velocity performance of the mobile robot would remain stable. Additionally, the control system parameter was adjustable with a graphic interface, which makes the robot suitable for operating with a variable load.

### 6.2. Higher-Level Control Results

**Figure 20:**
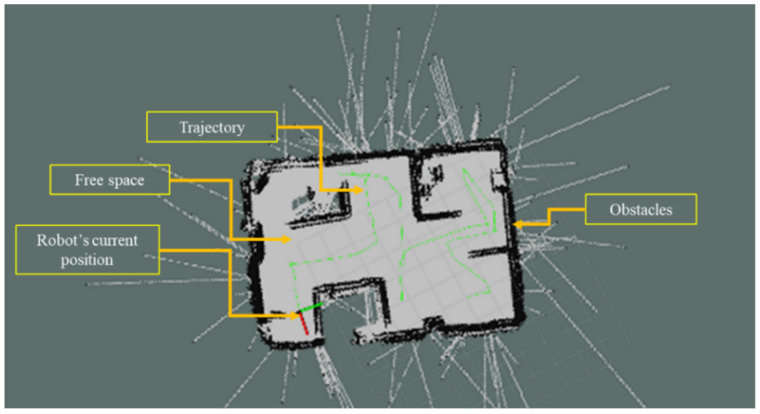
SLAM performance with MotionBot.

In the higher computer, the software architecture was adequate to perform a successful SLAM, although lots of error lines can be found outside the boundary shown in Figure 20. These mainly occurred due to sensor noise and reflections from different light sources. Further noise reduction algorithms can be developed for future research. Additionally, the LiDAR sensor has less effectiveness while passing through a glass or mirror. This phenomenon can be avoided by using more precious sensors or 3D camera sensors.

**Figure 21:**
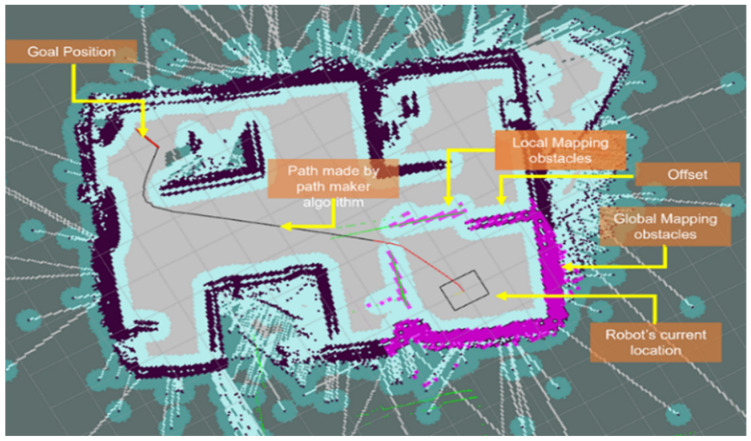
ROS autonomous navigation with MotionBot.

For the navigation architecture, the software prosperously made a path to the goal, avoiding all known and unknown obstacles. Thus, it can perform automatic navigation inside an indoor environment successfully. Figure 21 shows a performance of autonomous navigation of our mobile robot. To start the navigation, the initialization of the robot’s current location should be input with the ROS RVIZ interface and, with some iteration, the robot can localize itself perfectly. Then, with the help of the RVIZ interface, or by directly commanding the goal pose, autonomous navigation can be initiated.

**Figure 22:**
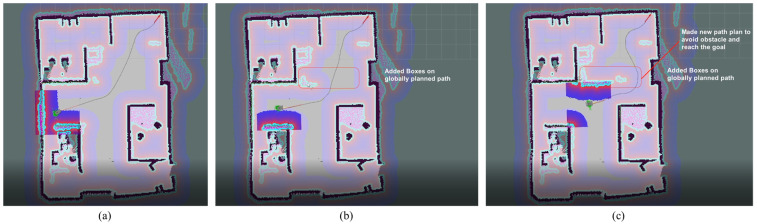
(**a**) Global path planner without considering local obstacles; (**b**) Added and detected obstacles on the Global path; (**c**) Correction of Global path to avoid obstacles.

The ability to avoid sudden obstacles, such as a human or unknown object, is also checked with the experimental mobile robot. In Figure 22, it is shown that the robot creates the global path to reach the goal according to the global map. However, as soon as obstacles are detected on the path, the local path planner adjusts the global path to avoid that obstacle and reach the goal.

**Figure 23:**
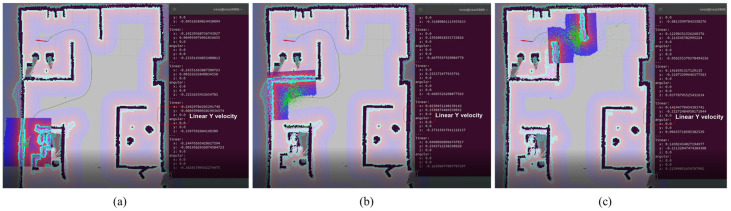
(**a**) Path planning in linear X direction; (**b**) path planning in linear Y direction; (**c**) path planning in both linear X and Y direction.

Path planning that considers lateral motion is also checked. Figure 23 shows a successful implication of the linear X and Y axis directional path planning, allowing the experimental mobile robot to take the shortest path to reach the goal.

## 7. Conclusions

The present study aimed to design and develop an omnidirectional mobile robot, which combined the characteristics of both an Autonomous Mobile Robot and an Automated Guided Vehicle. The results obtained from the practical operation of the ‘MotionBot’ robot, as discussed in previous sections, demonstrated the reliability, improvement, and effectiveness of the proposed techniques. The focus of the study was on enhancing the lower-level control through feedback and feedforward controllers to optimize vibrations and increase stability through a low computational cost. Additionally, the robustness of the robot was considered since it was expected to operate in different environments with different loads. A study and analysis of robustness was conducted, and the results confirmed its adequacy.

In order to enhance the sensing capabilities of a robot, a fusion of three LiDAR data was executed to improve the accuracy of localization and positioning. The performance of single LiDAR and multiple LiDAR using G-mapping SLAM was evaluated to increase mapping accuracy in unknown environments. The robot successfully reached the goal point while avoiding obstacles in a dynamic environment. A user-friendly GUI was developed using LabVIEW software. However, future research could be conducted to reduce LiDAR noise, address the wheel slip ratio problem, and implement object recognition and tracking technologies. The utilization of OpenCV and TensorFlow can enable the robot to analyze objects, such as human bodies, and follow them using object-following algorithms. The potential for further improvement, leveraging the capabilities of the ROS platform, holds promise for the logistics and courier industries.

## Figures and Tables

**Table 1:** Robot’s kinetic model variable and definition.

Variable	Definition
$\dot{Y_{r}}$	Instantaneous longitudinal velocity of the robot
$\dot{X_{r}}$	Instantaneous lateral velocity of the robot
$\dot{\theta_{r}}$	Angular velocity of the robot
$\dot{Y_{Wi}}$	Instantaneous longitudinal velocity of the i wheel
$\dot{X_{Wi}}$	Instantaneous lateral velocity of the i wheel
${\dot{\theta }}_{Wheel}$	Angular velocity of the wheel along $X_{Wi}$ axis (pitch axis)
${\dot{\theta }}_{Rot}$	Angular velocity of the wheel along $Z_{Wi}$ axis (yaw axis)
${\dot{\theta }}_{Roller}$	Angular velocity of the Roller when it contacts the ground
$\gamma_{i}$	Rotation angle between the i wheel frame and the roller frame
$\alpha_{i}$	Angle between robot main frame and the i wheel frame
$L_{w}$	Distance between robot coordinate and i wheel along x-axis
$L_{l}$	Distance between robot coordinate and i wheel along y-axis
R	Wheel radius
r	Roller radius
CoM	Center of mass of the robot

**Table 2:** Wheel and roller angular parameter and their values.

Symbol	${\mathrm{Wheel}}_{1}$	${\mathrm{Wheel}}_{2}$	${\mathrm{Wheel}}_{3}$	${\mathrm{Wheel}}_{4}$
$\alpha_{i}$	$\frac{\pi }{6}$	$\frac{5\pi }{6}$	$\frac{7\pi }{6}$	$\frac{11\pi }{6}$
$\gamma_{i}$	$-\frac{\pi }{4}$	$\frac{\pi }{4}$	$-\frac{\pi }{4}$	$\frac{\pi }{4}$

(Note: $L\cdot \cos\left(\alpha_{i}\right)=L_{w}$ // $L\cdot \sin\left(\alpha_{i}\right)=L_{l}$).

**Table 3:** Lower-level control system variable and definition.

Variable	Definition
$J_{n}$	Moment of inertia (0.0073969)
$B_{n}$	Friction constant (0.43571)
s	Output variable for Laplace transform
$\omega_{fb}$	Feedback band width
$C_{fb}$	Feedback control
$C_{ff}$	Feedforward control
$\omega_{ff}$	Feedback band width
ζ	Damping ratio
$\omega_{Q}$	Q-filter band width

## Data Availability

This study did not report any data.

## Abbreviations

The following abbreviations are used in this article:

AMR	Autonomous Mobile Robot
AGV	Automated Guided Robot
ROS	Robot Operating System
DOB	Disturbance Observer
CNC	Computer Numerical Control
IMU	Inertial Measurement Unit
CoM	Center Of Gravity
USATR	Universal Synchronous/Asynchronous Receiver/Transmitter
SLAM	Simultaneous Localization And Mapping
Lidar	Light Detection And Ranging
URDF	Unified Robot Description Format
DoF	Degree Of Freedom
GPS	Global Positioning System
RBPF	Rao-Blackwellized Particle Filter
AMCL	Adaptive Monte Carlo Localization
YAML	Yet Another Markup Language
PSO	Particle Swarm Optimization
RRT	Rapidly Exploring Random Tree

## Appendix A. Symbol and Definition

This appendix consists of a list (Table A1) with all the symbols used in this paper with their definitions.

**Table A1:** List of symbols and their definition.

Variable	Definition
$\dot{Y_{r}}$	Instantaneous longitudinal velocity of the robot
$\dot{X_{r}}$	Instantaneous lateral velocity of the robot
$\dot{\theta_{r}}$	Angular velocity of the robot
$\dot{Y_{Wi}}$	Instantaneous longitudinal velocity of the i wheel
$\dot{X_{Wi}}$	Instantaneous lateral velocity of the i wheel
${\dot{\theta }}_{Wheel}$	Angular velocity of the wheel along $X_{Wi}$ axis (pitch axis)
${\dot{\theta }}_{Rot}$	Angular velocity of the wheel along $Z_{Wi}$ axis (yaw axis)
${\dot{\theta }}_{Roller}$	Angular velocity of the Roller when it contacts the ground
$\gamma_{i}$	Rotation angle between the i wheel frame and the roller frame
$\alpha_{i}$	Angle between robot main frame and the i wheel frame
$L_{w}$	Distance between robot coordinate and i wheel along x-axis
$L_{l}$	Distance between robot coordinate and i wheel along y-axis
R	Wheel radius
r	Roller radius
CoM	Center of Mass of the robot
$J_{n}$	Moment of inertia (0.0073969)
$B_{n}$	Friction constant (0.43571)
s	Output variable for Laplace transform
$W_{fb}$	Feedback band width
$C_{fb}$	Feedback control
$C_{ff}$	Feedforward control
$W_{ff}$	Feedback band width
ζ	Damping ratio
$W_{Q}$	Q-filter band width
$e_{i}(k)$	Uncorrelated observation errors
$L_{pi}$	Jacobian matrix of observation model with respect to landmarks
$L_{v}$	Jacobian matrix of observation model with respect to robot odometry
$L_{i}$	Observation matrix that relates to the sensor output
$z_{i}$	The state vector x(k) when observing $i^{th}$ landmark
